# Fatal Course of a Male Newborn with Double Duodenal Atresia

**DOI:** 10.1055/s-0039-3400488

**Published:** 2020-02-07

**Authors:** Ahmed Elrouby, Ahmed Koraitim

**Affiliations:** 1 Department of Pediatric Surgery, Faculty of Medicine, Alexandria University, Alexandria, Egypt

**Keywords:** double, duodenum, atresia, abdominal cyst

## Abstract

Multiple point duodenal atresia is an extremely rare condition with atretic segments in either two or three sites of the duodenum. We report a newborn male patient who presented to our institution with bilious vomiting, nonpassage of meconium, mild abdominal distension, and a palpable epigastric abdominal mass ∼1 × 1 cm. A faint double bubble was found on abdominal X-ray. On exploratory laparotomy, a duodenal cyst due to double duodenal atresia was found and a typical diamond-shaped duodeno-duodenostomy was created. A postoperative contrast study revealed passage of the contrast media into distal intestine. However, the patient died 2 weeks later due to uncontrolled sepsis and pneumonia. Despite the fact that multiple-point duodenal atresia is a rare condition, it should be considered as a differential diagnosis to avoid missed pathology.

## Introduction

Multiple duodenal atresia is an extremely rare condition that may be either double or triple. To our knowledge, only 24 papers in the English literature reported this anomaly. Of these, two patients had triple duodenal atresia and the remainder had a double duodenal atresia. 1

Duodenal atresia is thought to occur by failure of embryological recanalization. However, a multiple atresia is difficult to explain with this theory. Yoshida and Migita described it as a result of a malrotation that causes a twist and results in pressure necrosis and atresia in more than one point. 2

This rare deformity has different presentations ranging from neonatal intestinal obstruction in case of complete atresia to infantile presentation with incomplete intestinal obstruction. This is associated with or without a palpable abdominal mass. 3 This anomaly can be associated with other conditions as Zamfir et al reported a case of double duodenal atresia associated with Cri du Chat (cat cry) syndrome. 4 Also this anomaly may be associated with peptic ulcer. 3

## Case Report

A male newborn (37 weeks of gestation; 2.43 kg) presented with bilious vomiting. Clinical examination revealed mild epigastric distension with the passage of mucous on digital rectal examination. A palpable epigastric firm mass measuring 1 × 1 cm was found. General examination revealed severe respiratory distress due to meconium aspiration pneumonia with associated signs of sepsis; however, there was no sign of peritonitis. Meconium aspiration pneumonia was diagnosed after reviewing the records of delivery that revealed meconium-stained amniotic fluid in association with immediate postnatal respiratory distress.

**Fig. 1:**
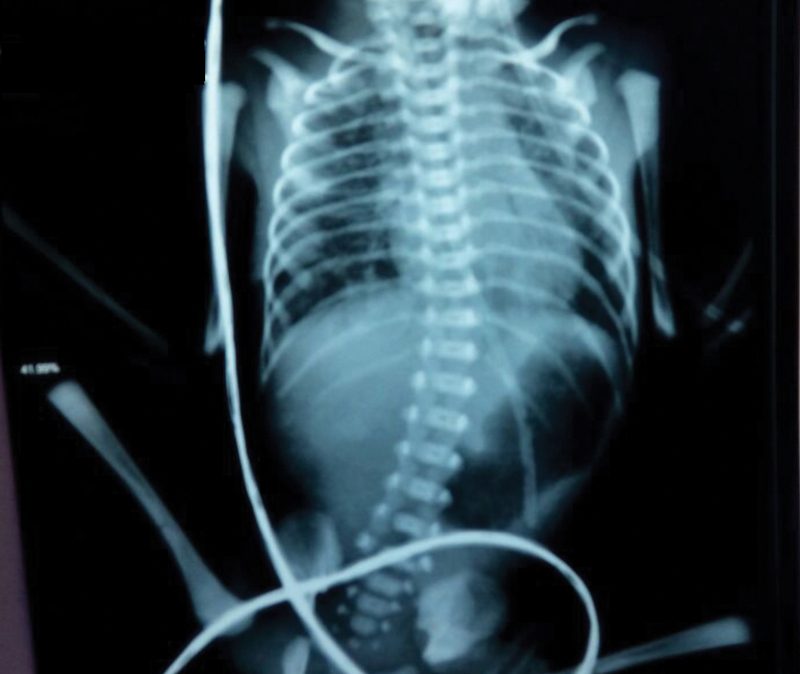
PXR abdomen standing showing hugely dilated stomach and first part of the duodenum (double bubble sign).

Laboratory investigations revealed hypochromic microcytic anemia with moderate leukocytosis in association with mild hypokalemia and metabolic alkalosis. Hanging abdominal X-ray showed a hugely distended stomach with a faint double bubble sign and absent gas distribution in the remaining abdomen as shown in Fig. 1 . Also, plain X-ray (PXR) chest revealed patches of consolidation and areas of hyperinflation that raised the suspicion of meconium aspiration pneumonia.

The mother had an antenatal ultrasound that revealed full stomach in association with polyhydramnios.

**Fig. 2:**
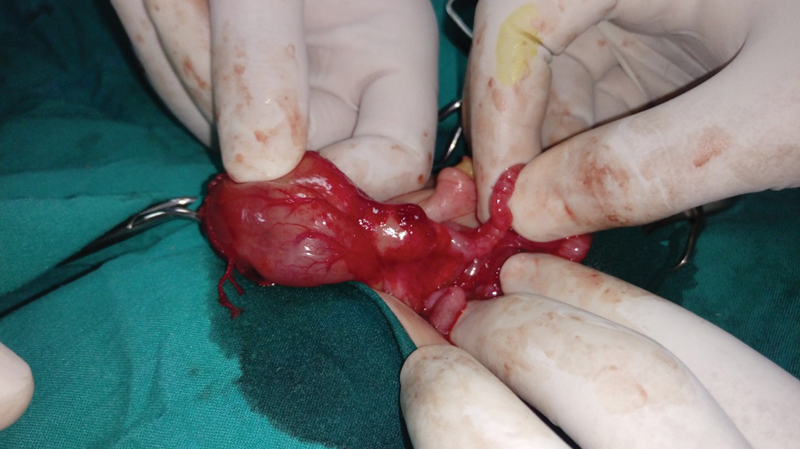
Intraoperative double duodenal atresia with duodenal cyst in between.

**Fig. 3:**
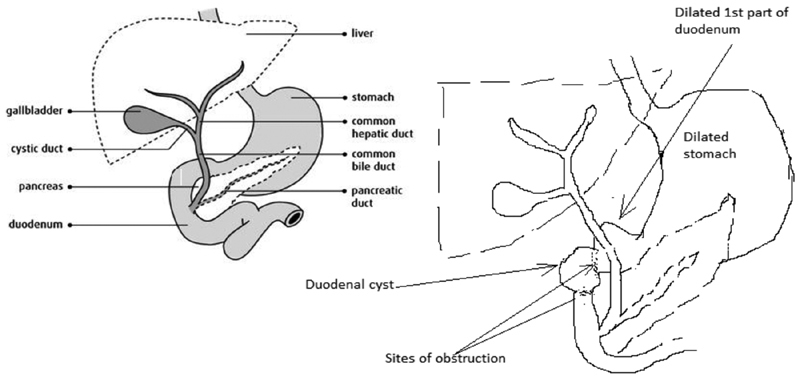
Illustration showing the duodenal pathology.

After stabilization of the septic patient, a right supraumbilical explorative laparotomy was performed. We performed a Kocher maneuver and found a cyst between the first and the second part of the duodenum as shown in Figs. 2 and 3 .

A longitudinal duodenotomy distal to the cyst was performed and the ampulla of Vater was identified. We advanced a 6-inch French tube through this incision into the distal intestine with injection of warm saline to exclude further distal intestinal obstructions. Then transverse duodenotomy proximal to the cyst was applied and we found a duodenal diaphragm at the proximal end of the cyst causing partial duodenal obstruction; this diaphragm was resected. Another complete duodenal atresia was found at the distal end of the cyst that explained the development of this cyst between a proximal partial obstruction and a distal complete obstruction. This cyst was bypassed by a diamond-shaped duodeno-duodenal anastomosis between the transverse duodenotomy proximal to it and the longitudinal duodenotomy distal to it.

**Fig. 4:**
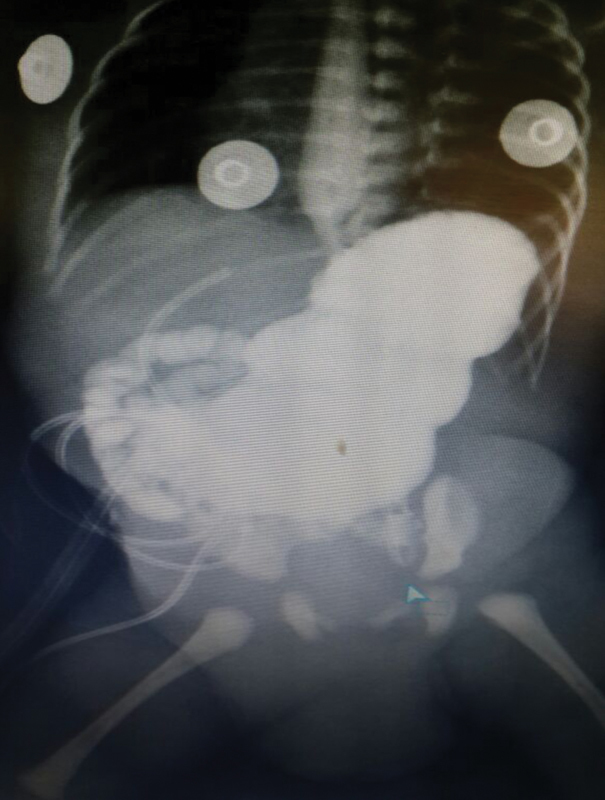
Postoperative contrast study with the dye passing into the small intestine.

Stool was passed on the 5th postoperative day; however, the child still had bilious reflux, so we performed a contrast passage study, which showed that the contrast passed to the distal intestine freely. However, with a remaining proximal dilatation the decision to delay oral intake was taken ( Fig. 4 ).

Unfortunately, the baby progressively deteriorated due to the meconium aspiration pneumonia that was resistant to antibiotic treatment. The condition was associated with severe uncontrollable sepsis that was progressed to septic shock, disseminated intravascular coagulation (DIC), and multiorgan failure that ended with death of the child 15 days postoperatively

## Discussion

Double duodenal atresia is an extremely rare condition occurring in 1:5,000 to 1:10,000 live births; Stringer et al published a series of four patients and the postoperative survival was reported in three cases and only one case died due to the associated congenital heart disease. 5 Also, along his 20-year experience, Grosfeld and Rescorla described only three patients with double duodenal atresia. 6

It usually occurs as a sporadic congenital anomaly and only two reports documented its presence in siblings. 7

Besides the previously mentioned presentations, double duodenal atresia may present postoperatively after the treatment of a first point duodenal obstruction by intestinal obstruction, perforation, and/or abdominal cyst. This usually develops if the patency of the distal intestine was not ensured during the first operation. 5 8

In our case, a cystic dilatation between the first and the second part of the duodenum was found during operative exploration. This is consistent with other similar studies discussing the same topic like that one belonging to Stringer et al, which was published in 1992. 5

Endoscopic incision of double duodenal webs has been advocated by some surgeons as a new treatment modality in infants presenting with partial double duodenal diaphragms. However, it is not feasible in newborns because a minimum weight of ∼8 kg is mandatory for endoscopic intervention as reported by Barabino et al. These authors recorded a successful endoscopic release of duodenal web in a 11-month-old baby (8 kg). 9 Another study was conducted by Bittencourt et al who documented 9 to 12 months as a minimum age for endoscopic intervention of infantile duodenal membranes. 10 Finally, an endoscopic dilatation and partial resection of a duodenal web were reported by Beeks et al in a 15-month-old patient (8 kg). 11

Reviewing the records of our patient during delivery revealed that delayed cord clamping was not done in this condition; this may be the cause of hypochromic microcytic anemia. Our patient died 2 weeks postoperatively from meconium aspiration pneumonia that was resistant to antibiotic treatment and associated with uncontrollable sepsis. The condition progressed to septic shock, DIC, and multiorgan failure that ended with death of the child. Surgical exploration and anastomosis are usually curative in all cases. Mortality is only known due to other causes, such as associated cardiac malformations or pneumonia as in our patient. 6

## Conclusion

Double duodenal atresia is a rare condition. Operative detection of one atresia does not preclude the presence of further distal one, so thorough examination of the distal intestine with a tube and warm saline injection in case of intestinal atresia is mandatory to exclude other distal obstructing lesions.

## Notes

New Insights and the Importance for the Pediatric Surgeon Double duodenal atresia is very important to be known.
